# Red Wine Colloids: Advances in Characterization and Strategies for Colloidal Stability

**DOI:** 10.1021/acs.jafc.6c01951

**Published:** 2026-05-01

**Authors:** Sara Zanella, Andrea Curioni, Matteo Marangon

**Affiliations:** † Department of Agronomy, Food, Natural Resources, Animals and Environment (DAFNAE), University of Padova, 35020 Legnaro, Italy; ‡ Interdepartmental Centre for Research in Viticulture and Enology (CIRVE), University of Padova, 31015 Conegliano, Italy

**Keywords:** colloidal, red, wine, colloids, advances

## Abstract

Understanding the colloidal and macromolecular composition of red wine is a critical yet complex topic in modern enology. Wine colloids significantly influence sensory and physicochemical properties, including mouthfeel, astringency, color stability, and aging potential. This review summarizes recent advances in understanding how wine colloids form, evolve, and can be managed during winemaking. The effects of traditional practices, such as clarification and filtration, as well as emerging targeted approaches (e.g., enzymatic treatments), are discussed. Relevant literature was identified through PubMed, Scopus, and Google Scholar, focusing on studies related to red wine colloids. The analysis highlights the absence of standardized methods for achieving complete colloidal stabilization, largely due to the complexity and dynamic nature of colloidal interactions in wine. Despite progress in this field, further advances will require improved models that more accurately describe colloid behavior, supporting the development of effective stabilization strategies to preserve red wine quality.

## Graphical abstract

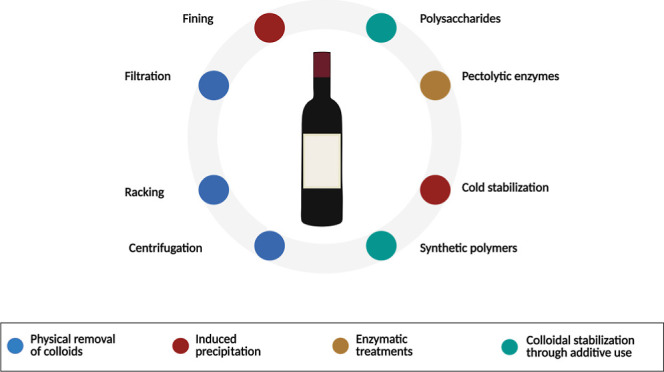

## 1 Scientific Context

Although inherently challenging to study, red wine colloids have gained increasing attention in recent years due to their fundamental impact on wine stability, quality, and sensory perception. Red wine, as a hydroalcoholic solution, serves as a continuous medium in which various macromolecules are present. Among these, principally condensed tannins, polysaccharides, and proteins can interact through chemical and physical forces, leading to the formation of different particles, which remain in dispersion as colloids.^1^

Wines typically contain multiple populations of colloids that differ in composition and, as a result, in size. These colloids range from individual chemical species just a few nanometers in diameter, indicated as polymeric, such as proteins, polysaccharides, or condensed tannins, to larger complexes measuring up to approximately 200 nanometers, in which the above-mentioned polymeric colloids can be found in an aggregated form,^2–5^ giving rise to a dispersed system. Depending on the nature and physicochemical characteristics of the phases of this dispersed system, colloidal dispersions in wine may remain stable over time or become unstable, leading to the formation of larger particles able to scatter the light, resulting in wine turbidity.^2^ This turbidity in wine is generally considered a fault by consumers, as it affects the visual appeal and suggests inaccurate winemaking. However, colloidal aggregation can have broader implications beyond appearance, influencing key aspects of wine quality.^6^ Indeed, ensuring colloidal stability is essential not only to maintain clarity over time but also to preserve the integrity and sensory qualities of the wine. It is well-known that condensed tannins, polysaccharides, and proteins play a direct role in defining key sensory attributes of red wine, such as color, astringency, mouthfeel, and overall flavor profile. These same macromolecules are also the main constituents of red wine colloidal particles, which contribute to wine quality since they include pigments resulting from the reaction between anthocyanins and condensed tannins (polymeric pigments) and possibly interact with volatile compounds. When destabilized, the loss of such colloids can lead to color loss and a diminished aromatic expression; conversely, their stability helps maintain the wine’s color and sensory complexity.^7^ Various enological approaches, such as fining, filtration, and enzyme addition, have been explored to manage colloidal instability while preserving wine quality.^6,8–10^ However, selecting the most suitable approach remains a challenge, as treatments must effectively stabilize wine without negatively impacting its sensory attributes.

Colloidal particles in wines are inherently heterogeneous, as their constituents can originate from various sources, including natural components like grapes, yeast, and bacteria, as well as additives and fining agents used by the winemakers. Moreover, their quantity and characteristics can vary depending on factors such as climate, vinification techniques, and wine storage conditions.^11–15^ Therefore, the detailed investigation of these particles has always been challenging. Researchers face several obstacles, including the complex interactions between colloid-forming molecules, their variability across different winemaking scenarios, and the analytical difficulties in studying them without disrupting their native molecular interactions. Recent advancements in analytical techniques, such as asymmetrical flow field-flow fractionation (AF4) coupled with multidetection systems, have significantly enhanced our ability to study wine colloids and track their evolution during aging.^1,2,4,5^ Additionally, dynamic light scattering (DLS) and nanoparticle tracking analysis (NTA) are widely used to investigate the physical properties of colloidal particles in wine; these techniques are particularly useful for analyzing particle size distribution at the nanometer scale.^16–19^ More recently, electrophoretic light scattering has been applied to assess the electrical potential at the particle−liquid interface, which determines the electrostatic repulsion or attraction between colloidal particles and can now be determined instrumentally.^15,20,21^

Despite these advances, the study of red wine colloids has progressed more slowly than other areas of wine science, likely due to their inherent complexity and the methodological challenges that they present. This is reflected in the relatively limited number of publications specifically focused on the structure, behavior, and stability of red wine colloids: the first appeared in 1991, and only 34 relevant studies have been published since then, according to a Scopus search performed on April 30, 2025. The search used the query *“red wine” AND (colloids OR particles) and (stability OR aggregation)*. Publications from 2025 were excluded, as the year was still in progress at the time of writing. All document types were initially included in the search, and irrelevant records were subsequently excluded after screening titles and abstracts and, when necessary, full texts, based on their lack of relevance to the topic of colloidal stability and aggregation in red wine. After manual screening, 34 articles were retained for analysis. The heatmap of Figure 1 summarizes the evolution of the main author keywords associated with this research area over time. Although relevant articles were published earlier, article keywords were consistently available only from 1997 onward. These keywords were extracted from the selected articles and grouped according to thematic similarity (e.g., colloids, proteins, polyphenols, etc.), and their frequency of use was tracked by year of publication. Two main aspects clearly emerge from the analysis. First, core keywords such as colloids, proteins, polysaccharides, and polyphenols appear more frequently throughout the years, highlighting their central role in the study of colloidal phenomena in red wine. Second, the frequency of occurrence of the keywords used markedly increased over time, with a peak observed between 2020 and 2024, indicating a renewed and growing scientific focus on the topic. Moreover, keywords directly related to color, such as color, pigments, and anthocyanins, are largely absent in earlier years and only start to appear with greater frequency in the past decade, suggesting that the scientific community has only recently begun to explicitly address the relationship between colloidal phenomena and color stability.

**Figure 1:**
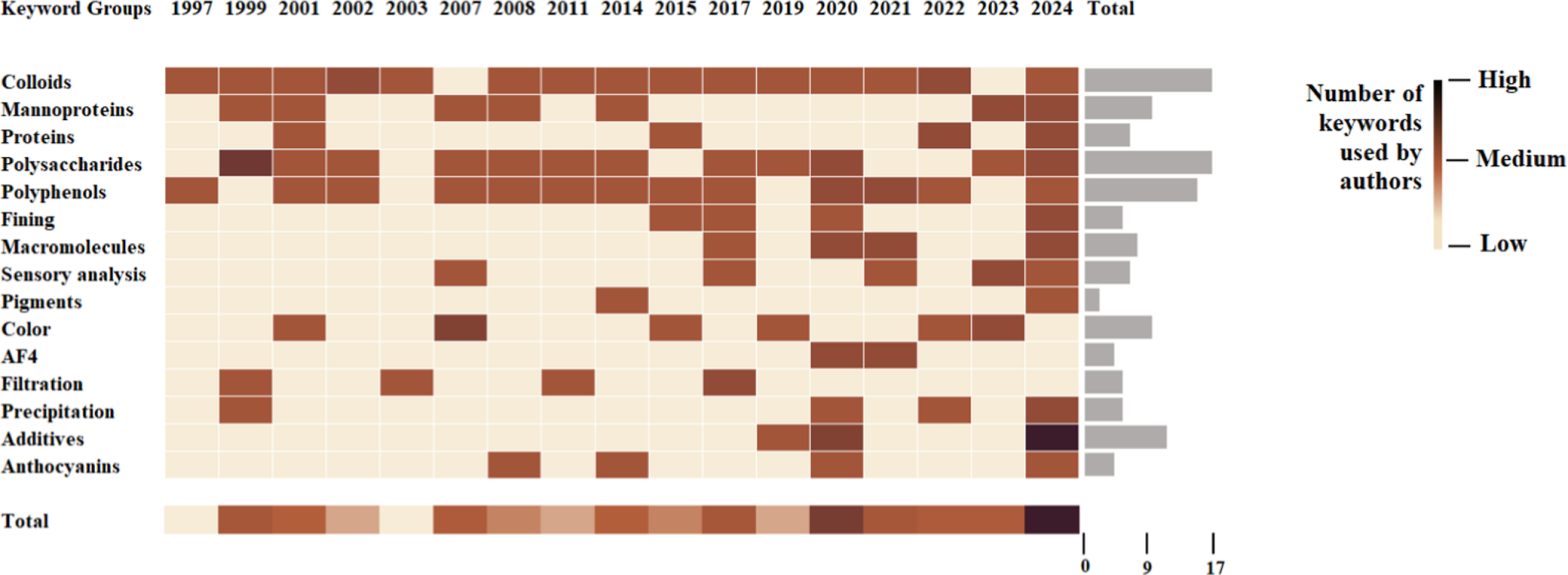
Heatmap showing the temporal evolution of the most frequently occurring author keywords in scientific publications focused on red wine colloids, published between 1997 and 2024. These publications were selected from a broader data set retrieved via Scopus according to a search conducted on April 30, 2025.

Historically associated with instability, particularly in relation to protein haze and sediment formation, colloids are now increasingly recognized as functional elements that modulate other wine key attributes such as mouthfeel, astringency, color retention, and aging potential (Figure 1). Generally speaking, over the past two decades, research has progressively shifted from a reductionist view focusing on the study of isolated compounds to a more realistic perspective on wine colloids, trying to understand their actual nature and dynamics.

The literature included in this review was identified through searches conducted in PubMed, Scopus, and Google Scholar to ensure broad and comprehensive coverage of the topic. Searches were performed using combinations of the following keywords: “red wine”, “colloids”, “particles”, “stability”, “aggregation”, “turbidity”, “polysaccharides”, “proteins”, and “phenolic compounds”. No strict lower limit for the publication year was applied; however, priority was given to recent studies and those directly addressing the composition, formation, stability, or management of red wine colloids. Scopus was specifically used for the evaluation of publication trends and the generation of the keyword heatmap, due to its comprehensive and standardized metadata, which facilitate quantitative analysis of author keywords.

This review examines the current state of knowledge on red wine colloids with a focus on their composition, formation, and management throughout the winemaking process. It highlights key research trends, identifies persistent knowledge gaps, and evaluates the impact of traditional enological practices, such as clarification and filtration, in light of emerging, more targeted techniques. Finally, the review outlines future research priorities aimed at bridging a fundamental understanding with practical applications in modern winemaking.

## 2 Origin of Colloids in Red Wine

Although the idea that colloidal particles dispersed in wines originate from the interaction of different types of (macro)­molecules is not new, only recently has it been clearly demonstrated that these particles can comprise proteins, polysaccharides, and phenolic compounds, whose relative quantity and type modulate their characteristics. Most of these molecules originate from grape berries, especially skins and pulp, while others, such as mannoproteins, derive from yeast cell walls and are released during fermentation and aging on lees.^7^ In addition, grape seeds and, to a lesser extent, grape stems can also contribute significantly to the pool of extracted phenolic compounds, particularly condensed tannins, depending on grape variety and winemaking conditions.^22^ A series of factors affect the concentration and composition of all the colloid-forming molecules in grapes, beginning with the grape variety itself.^23^ Additionally, climatic conditions and terroir-related factors play a major role in shaping the composition of grapes and, consequently, the colloidal profile of the resulting wine.^24,25^ Grape maturity is another key factor influencing the composition of wine colloids, as the progressive degradation and structural modification of cell wall components during ripening enhances the extractability of polysaccharides, phenolic compounds, and other macromolecules that contribute to the colloidal fraction of red wine.^26^ Besides the intrinsic compositional differences derived from the grapes, several external variables acting during vinification, aging, and storage play a significant role in both the extraction and the removal of colloid-forming molecules, thus affecting the formation and evolution of colloidal structures. In particular, winemaking practices such as skin maceration modulate the extraction of specific wine components, thereby affecting red wine colloids.^2,12,27,28^ Moreover, other enological practices such as aging on lees,^29^ barrel aging,^30^ and the use of specific additives (e.g., mannoproteins, enological tannins, and/or polysaccharides) can enrich the wine with compounds,^31–33^ which are reported to play a role in the formation of colloidal structures.^1^ Conversely, practices such as protein or bentonite fining, as well as filtration, can result in the partial or complete removal of some compounds involved in wine colloidal particles.^34,35^ These facts should be considered by winemakers when the colloidal state and stability of the wine are important determinants of quality.

### 2.1 Molecules in Colloidal Particles: Phenolics, Polysaccharides, and Proteins

Recently, the presence of polyphenols, proteins, and polysaccharides in red wine colloidal particles has been confirmed.^1,3^

Polyphenols are the dominant class of compounds in red wine, including flavanols present as monomers (e.g., catechins) as well as oligomeric and polymeric forms corresponding to proanthocyanidins (PAs), commonly known as condensed tannins. In addition to flavanols, other phenolic classes such as anthocyanins, flavonols, and phenolic acids are also present in red wines. These compounds primarily derive from grape skins and seeds, from which are extracted during maceration in the early stages of winemaking.^12,36^ Additional phenolics, such as ellagitannins, can be extracted from oak barrels during aging, their extraction being influenced by time, temperature, and alcohol content.^37^ A recent study measured the total phenolic content (TPC) of 110 Italian monovarietal red wines, reporting values ranging from approximately 1065 to 3053 mg/L.^38^ Comparable findings have been documented internationally, using non-Italian wines,^39–41^ highlighting that the variations in TPC content largely reflect differences in grape cultivar, terroir, and vinification methods. Several studies consistently emphasize polyphenols as critical contributors to the sensory attributes of red wine, because they clearly affect color intensity, astringency, bitterness, mouthfeel, as well as the antioxidant capacity.^34,42,43^ Apart from their importance as direct contributors to those characteristics, which have been studied for a long time, the involvement of polyphenols in colloidal particle formation in red wines is now almost certain. As a matter of fact, one of the main features of polyphenols, particularly tannins, is their ability to interact with biopolymers, which in wine include both polysaccharides and proteins. This can occur with different mechanisms involving chemical bonds (oxidative reactions and hydrogen bonding), hydrophobic interactions, which seem to be particularly important, and also enzymatic (oxidases) reactions.^44^ Therefore, the tendency of polyphenols to form complexes with polysaccharides and proteins present in wines can certainly be expected. However, the specific types of interactions occurring in wine are not yet completely understood, although recent evidence indicates that both covalent and non-covalent bonds can form between proteins and polyphenols.^3^

Polysaccharides are present in red wines at concentrations up to 2 g/L.^7,45^ According to their origin, polysaccharides can be classified into four main categories: (i) those deriving from grape skins (PRAGs, polysaccharides rich in arabinose and galactose); (ii) mannoproteins, released by yeasts; (iii) glucans and other polysaccharides, originating from fungal infections like those of*Botrytis cinerea;*and (iv) polysaccharides added to the wine as technological aids.^7,28^ Throughout maceration, numerous grape-derived polysaccharides undergo enzymatic degradation, releasing undegradable fragments which remain in the wine.^7,46^ In contrast, yeast mannoproteins (MPs), which are continuously released during both fermentation and aging on lees, are more resistant to degradation.^15,28,47,48^ Due to their abundance and chemical characteristics, mannoproteins seem to play a crucial role in wine colloidal stability and sensory properties, enhancing mouthfeel, modulating tannin reactivity, and improving wine stability.^7^ However, certain technological interventions can reduce wine polysaccharide content, particularly when these compounds are associated with colloidal particles (see Section 3.1 for a detailed discussion on microfiltration).^49^

Several studies report on the interactions of polysaccharides with proteins and polyphenols in model solutions, trying to explain their effects in terms of wine stability, clearly demonstrating that these interactions occur. Certain polysaccharides can directly interact with proteins or polyphenols to induce precipitation, while others can delay such interactions in a pH-dependent manner.^50–52^ The precise mechanism on the basis of those interaction phenomena in the context of colloidal particle formation in wines is not known, although non-covalent interactions seem to be mainly involved.^53^

There is substantial literature supporting the role of the charge of polysaccharides in driving ionic or electrostatic interactions, thus affecting precipitation dynamics.^54^ Due to this charge, polysaccharides can establish ionic or electrostatic interactions with other macromolecules,^55^ as more recently confirmed by measuring zeta potential and particle size of protein–polysaccharide interactions.^20^ Another possibility would be the presence, in polysaccharides, of a polypeptide moiety, as occurs, for example, in yeast mannoproteins and in arabinogalactan peptides, which can be found in wine. This part of the molecule can virtually confer some hydrophobicity to the polysaccharide, thus allowing the onset of hydrophobic interactions with both proteins and polyphenols, giving rise to colloidal complexes with enhanced stability, as, for example, occurs with mannoproteins, which can stabilize tannins^56^ and also the colloidal coloring matter.^8^

The primary proteins present in red wines are similar to those found in whites, which have been studied in detail due to their involvement in haze formation in the latter.^23,51,57^ Most of these proteins belong to the Pathogenesis-Related (PR) proteins and are consistently found across all*Vitis vinifera*cultivars.^57,58^ They consist mainly of Thaumatin-like proteins (TLPs) and Chitinases, originating from grape berries.^59^ These proteins influence wine stability and, through interactions with polysaccharides and tannins, affect turbidity, color, and astringency.^20,60^ Historically, accurately quantifying proteins in red wines by the most common colorimetric methods has been difficult due to the presence of various interfering compounds and, in particular, polyphenols that are abundant in red wine.^61^ One of the most promising approaches involves protein precipitation with cold trichloroacetic acid/acetone, followed by dye-binding quantification using yeast invertase as a reference standard. Using this method, Smith and colleagues reported protein concentrations in aged Pinot noir wines ranging from 50 to 102 mg/L, with a mean value of 70 mg/L.^62^ In a more recent study conducted using the same method, protein concentrations ranging from 0 to 159 mg/L, with an average of 41 mg/L, were reported across several Italian red wines.^23^ However, the main problem for the dye-binding methods is the possible interference of the polyphenols covalently bound to the precipitated proteins, whose reactivity with the dye cannot be excluded, nor quantified. To avoid this problem, a possibility would be the quantification of the amino acids released by acid hydrolysis from ethanol-precipitated material. Indeed, using this system, protein concentrations in a range of red wines were reported, with values ranging from 23 to 380 mg/L.^61^

In the case of proteins, due to the presence of a large number of amino acids showing a broad spectrum of chemical characteristics, there are multiple potential mechanisms of interaction with other (macro)­molecules, including hydrophobic interactions and hydrogen and ionic bonds. These types of interactions, in general, are highly dependent on the characteristics of the environment, in particular, its pH, polarity, ionic strength, and temperature, which can also cause partial or total protein unfolding with changes in the level of exposure of the tannin binding sites. For example, it has been demonstrated that wine proteins of different hydrophobicities have different affinity for tannins, and this affinity is also affected by heating and disulfide bond reduction.^63^ Due to these characteristics, the involvement of proteins in the formation of colloidal particles in red wines is now almost certain.

### 2.2 Characterizing Wine Colloids

Understanding how the molecules presented in the previous section interact is essential, as the structure of red wine colloidal particles seems to depend on the concentration of those macromolecules and their ability to interact with one another.^2,7^

Colloids have often been investigated by isolating them by ultrafiltration or ethanol precipitation,^15,64–66^ which almost certainly alter their native state, impairing the understanding of the true structure and interactions of colloidal particles. Different chromatography approaches have been proposed to separate and analyze individual wine macromolecules as well as their colloidal assemblies, including size-exclusion chromatography, ion-exchange chromatography, and fast protein liquid chromatography. While these techniques provided key information on wine macromolecules (e.g., polysaccharides characterization), they led to the disruption and loss of colloidal structures stabilized by weak bonds.^64–66^ Also recently, for example, the identification of the components present in colloidal particles of red wines^15^ required preparation procedures that likely had an impact on their state, especially when considering the weak interactions involved in the stabilization of these structures. To overcome these limitations, simplified model systems have been widely adopted. DLS was used to investigate macromolecular interactions in model wines, providing evidence that polysaccharides can inhibit tannin aggregation and modulate particle size evolution^67,68^ or protein–tannin interactions.^69^ Measurements of zeta potential and turbidity were also used to infer colloidal stability, showing that colloids exhibit a strong pH-dependent ζ-potential within the wine pH range (3–4), a behavior that suggests acidity significantly influences their reactivity and stability.^15^ However, these techniques proved inadequate for revealing detailed structural information. Another approach involves the use of NTA for the characterization of red wine colloids, with a few promising studies emerging in recent years. Indeed, NTA proved suitable for quantifying native colloidal particles directly in wine, revealing shifts in particle size distribution associated with aggregation and detecting large aggregates through the light-scattering intensity. When combined with complementary techniques such as DLS and UV–vis spectroscopy, NTA helped relate particle size and abundance to macromolecular composition and interaction behavior.^16,70^

The recent introduction of AF4 has marked a major advancement, as it allows the separation and characterization of colloids under conditions closely resembling those found in wine. Unlike traditional chromatographic methods, AF4 operates without a stationary phase or the need for specific buffers or solvents, thereby preserving the chemical and physical integrity of the native colloidal particles. These can be fractionated according to their hydrodynamic size and precisely characterized using multidetection systems.^71^ Moreover, the separated colloidal fractions can also be collected and chemically analyzed to determine their composition.^1^

Initial research with AF4 primarily focused on individual colloid-forming molecules such as tannins and polysaccharides, aiming to optimize analytical conditions and separation parameters. This study used tannin fractions extracted from apples and polysaccharides from various red wines, simulating wine conditions during analysis.^72^ More recently, the same authors investigated the relationship between colloidal fractions and perceived astringency, further refining AF4 methodologies for wine colloid characterization.^73^

### 2.3 Structures and Functionality of Colloidal Particles in Red Wines

Colloidal aggregation in red wines has historically been linked to the well-known interaction between tannins and proteins. For decades, it was commonly believed that proteins would not be present in red wines because the extraction of large quantities of phenolic compounds during maceration would lead to their complete precipitation. Indeed, early models for colloidal aggregation in red wines incorporated this concept, as postulated by Saucier^74^ (Figure 2A). According to the first model, flavanol molecules extracted during maceration first interact with one another through hydrophobic interactions, forming colloidal flavanol particles. These particles can then either react with wine proteins via van der Waals interactions and precipitate or interact with polysaccharides to form stable solutions. Therefore, although the model did not account for the presence of free proteins in red wines, it has the merit of highlighting the involvement and importance of wine polysaccharides for the stabilization of colloidal particles.

**Figure 2:**
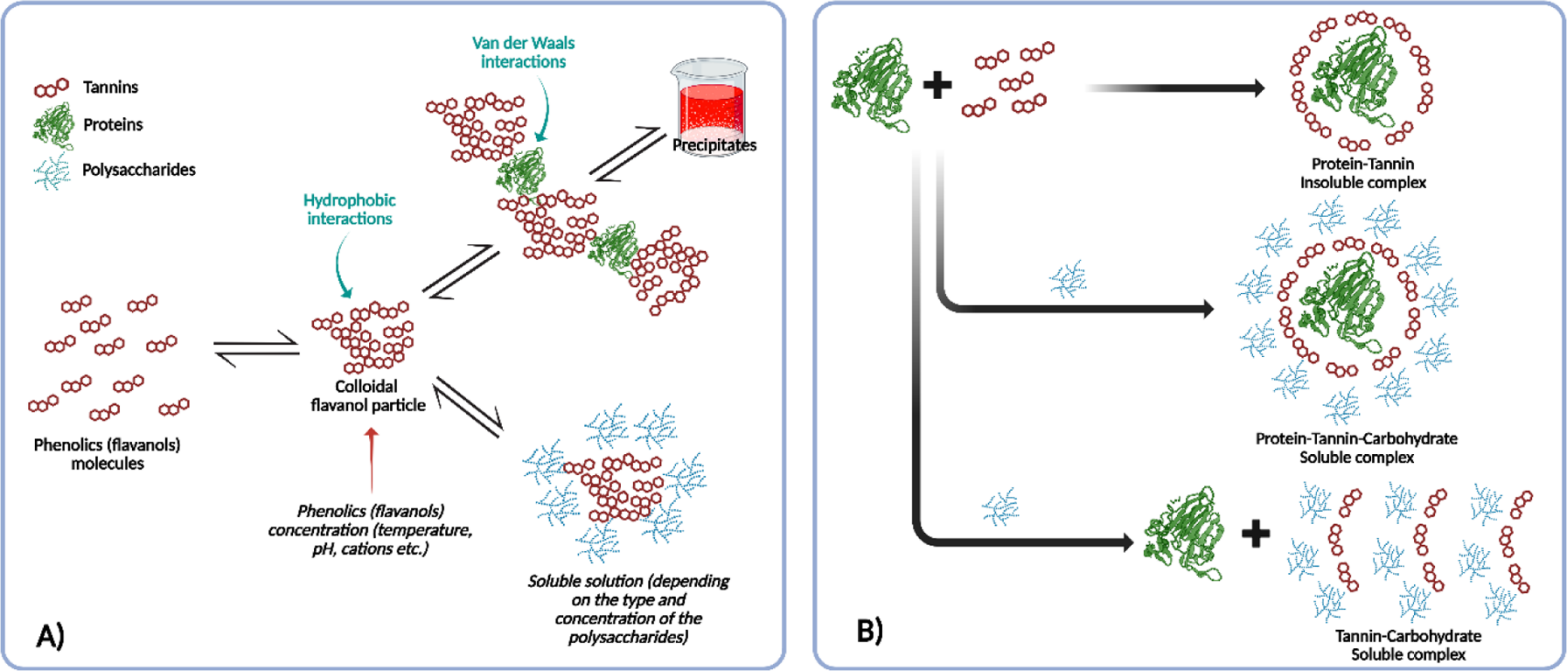
Early models of colloidal aggregation in wines. (A) Redrawn from Saucier.^74^ (B) Redrawn from Mateus.^69^

Almost a decade later, Portuguese researchers, using model systems, attempted to better understand how wine macromolecules interact to form different colloidal particles^69^ (Figure 2B). Using individual colloid-forming molecules, they hypothesized that tannins interacting with proteins result in a precipitate (insoluble complex), whereas tannins interacting with polysaccharides yield soluble colloidal particles. They also reported that, in some cases, not all proteins participate in precipitation with tannins; this behavior largely depends on the wine matrix and on the protein-to-tannin ratio. Indeed, their results indicate that proteins may exist in free form in red wines, while protein-tannin aggregates may also remain soluble when interacting with polysaccharides to form a ternary complex. These findings also shed light on the affinity of tannins for proteins, although the conclusions were drawn from relatively simple model systems rather than real wines.

As research progressed, it became clear that the molecular interactions in real wines are far more complex than those in model solutions, as numerous compounds and wine parameters can affect the reactivity of the individual colloid-forming molecules.

More recently, advances in research have allowed gathering of further data on the mechanism of red wine colloidal formation, especially when the AF4 technique was applied to samples produced under controlled winemaking conditions to analyze the composition of their colloidal particles in terms of the presence and quantity of interacting (macro)­molecules (proteins, polysaccharides, and polyphenols).^1^ Conversely to previous AF4 studies on red wine, the authors also considered the presence of proteins, which were analyzed by SDS-PAGE. No free proteins corresponding to those normally found in white wines were detected, even under strong denaturing conditions, suggesting that all proteins are covalently bound to tannins in the colloidal particles of the red wines analyzed. The overall results suggest that tannins and proteins form covalent sub-aggregates, which then interact with polysaccharides (including mannoproteins) through non-covalent forces. The ratio between these macromolecular components and the type of bonds they form plays a critical role in colloid stability.^1^

Further research by Marangon et al. examined 24 monovarietal Italian red wines, highlighting significant differences in colloidal structures across wines produced from different grape varieties. The study demonstrated that the quantity, size, and shape (compactness) of colloids are strongly linked to varietal characteristics. All wine colloids were shown to contain color-associated compounds (polymeric pigments) interacting with other wine components, including proteins and polysaccharides.^2^ These interactions contribute to the formation and stabilization of colloidal particles, as summarized in Figure 3.

**Figure 3:**
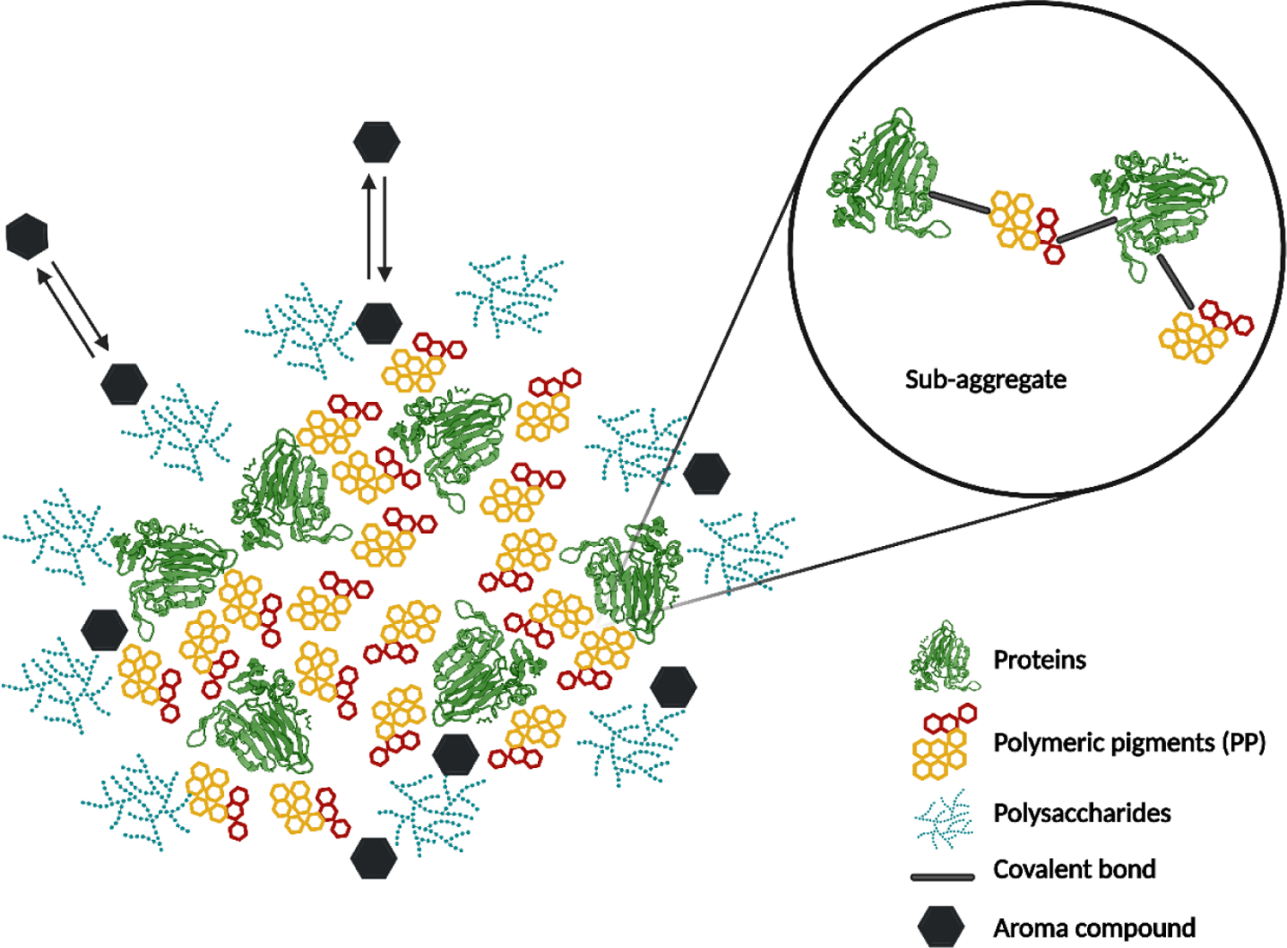
Red wine colloidal structure.

These findings support existing theories of colloidal formation, demonstrating that each component plays a distinct role in the process (Figure 3). In particular, tannins, especially those with the highest tendency to react with proteins (*T*_BSA_), can form covalent bonds with proteins, leading to the formation of small tannin–protein complexes (sub-aggregates). These sub-aggregates can be subsequently associated with each other through non-covalent interactions, resulting in the formation of larger colloidal particles. The quantity of *T*_BSA_ varies among monovarietal wines, emphasizing the impact of grape variety on colloidal structures of the resulting wine.^23^ In contrast, polysaccharides, particularly mannoproteins, are associated with these particles through non-covalent interactions, contributing to colloidal stability.^2,3,7,75^

Evidence suggests that colloidal instability can lead to several common issues, negatively impacting the wine’s sensory attributes, appearance, and overall consumer satisfaction.^6,9^ One such issue is haze or cloudiness, which happens when colloidal particles aggregate and grow, becoming large enough to scatter light and be visible to the naked eye. This phenomenon can further evolve, resulting in the precipitation of (colored) matter, detectable as sediment. Inevitably, both haze and sediment are generally considered undesirable by consumers, negatively affecting the wine-drinking experience.^32^ From a visual point of view, due to the involvement of polymeric pigments in colloidal structures,^2^ colloidal destabilization could lead to color precipitation, resulting in a worsening of wine color characteristics.

Beyond these interactions, colloids may also be associated with aroma compounds, further influencing the sensory properties of red wine.^2,3,7,76–78^ The loss of wine volatile compounds can be another adverse effect of colloidal particles’ precipitation. Indeed, it is known that colloids can adsorb small molecules, including volatile compounds, onto their surfaces. Therefore, the aggregation and precipitation of particles can result in the removal from solution of a portion of the aroma compounds associated with them. On the other hand, when the colloidal system of the wine is stable, aroma compounds associated with the particles through weak, reversible interactions can be retained in the wine and gradually released according to the equilibrium between bound and free components (Figure 3). However, interactions between volatile compounds and colloids are very complex and depend on several factors, including the type and concentration of the interacting molecules and the physicochemical conditions of the wine.^79,80^

The taste of red wine may also be affected by the type and concentration of its colloids. The effect of individual colloid-forming macromolecules on astringency and mouthfeel has been widely investigated,^60,75^ including through advanced methods for studying colloids under native conditions, such as AF4.^73^ Although the impact of their aggregation into colloidal particles, such as those described above, is not yet known, by looking at the models (Figures 2 and 3), it could be hypothesized that when phenolic compounds are part of a colloidal structure, their binding sites are occupied by interactions with proteins and polysaccharides. This may render them less reactive to salivary proteins, leading to a reduction in perceived astringency.^60,75^ If confirmed, modifications to the wine colloidal status through different winemaking practices could also have the potential to influence wine taste.

## 3 Strategies for the Management of Colloids in Red Winemaking

Effective management of colloids in red winemaking requires a comprehensive understanding of their formation mechanisms, evolution during aging, and relationship with various aspects of wine quality. This, in turn, requires an in-depth physicochemical characterization of colloidal particles to determine their composition, structure, and interactions with other wine components. At the same time, assessing colloidal stability is essential to anticipate and control colloid-related instabilities over time. As evidenced from the commentary of Figure 1, progress in the study of wine colloids lags behind other areas of wine science. Nevertheless, a deeper understanding of colloidal systems is crucial for developing effective, evidence-based strategies that help to preserve both the sensory and visual quality of red wines. In winemaking, practical management of the wine colloidal fraction is as important as a scientific understanding of its physicochemical properties. Assessing and ensuring colloidal stability often require a range of processing techniques designed to minimize the risk of colloidal destabilization during the lifespan of a wine. These techniques act on wine composition in different ways: some remove colloid-forming constituents or colloidal particles, others induce flocculation, and some aim to prevent further aggregation of wine components.^6,9^

The strategies that can impact the colloidal state of the wine with consequences on its stability can be classified into four main categories according to their mechanisms of action (Table 1):

1. The first category, physical removal of colloids, includes techniques aimed at the mechanical separation of suspended particles, primarily to enhance clarity and microbial stability. This includes filtration and ultrafiltration, racking, and centrifugation.
2. The second category, induced precipitation, promotes the removal of unstable compounds. This strategy can be targeted for the removal or alteration of specific wine components. This includes cold settling and fining.
3. The third category, enzymatic treatments, is for the targeted hydrolysis of macromolecular colloids (proteins and polysaccharides).
4. The fourth category, colloidal stabilization through additive use, includes strategies that inhibit colloidal growth and aggregation by incorporating compounds such as polysaccharides.

**Table 1:** Overview of Key Winemaking Techniques for Red Wine Colloid Management^a^

treatment	mechanism of action	winemaking application	effects on colloids	sensory and/or technological effects	refs
***I. Physical Removal of Colloids***					
racking	gravity separation	clarification of must and fermented wine, including removal of adjuvants after fining	tartrate crystals, large aggregates, and yeast removal	ineffective for removal of small colloids	^6,81,82^
centrifugation	centrifugal force separation	separation of particulates from young wines or after fining	tartrate crystals, large aggregates, and yeast removal	ineffective for removal of small colloids	^6^
dead-end filtration	retention of solids and microorganisms by porous filtration	removal of larger sediments (>1 μm)	ineffective for colloidal stabilization	possible impact on mouthfeel, aroma, and color stability	^81,85,100^
microfiltration		elimination of colloidal aggregates, yeast, and microorganisms (0.2–10 μm)	microbial stabilization and filtration of larger particles	possible flavor compounds stripping	^84–86,88,91^
ultrafiltration		removal of macromolecules and small colloids (0.01–0.1 μm)	reduction of the majority of the colloidal matter	organoleptic alteration for overclarification	^85–87^
***II. Induced Precipitation***					
cold stabilization	crystallization and precipitation	tartrate stabilization	destabilization of colloids and protein–polyphenol aggregates	loss of beneficial macromolecules (color, body, aroma)	^5,8,97^
animal and plant protein fining agents	flocculation and complexation with tannins and phenolic compounds	tannin softening in red wines with unstable colloidal pigments	enhancement of stability and clarification	haze minimization; reduction of astringency and color intensity	^81,99,100,102–104,106^
bentonite	adsorption of positively charged compounds	colloidal color stabilization and wine clarification	protein stabilization; reduction of polyphenols and pigments	possible impact on aroma and color	^6,106,112^
PVPP/PVP	phenolic adsorption	eliminating off-odors	fractionation of low-weight phenolics	removal of bitter compounds and browning precursors; possible color removal	^81,100,103,113^
***III. Enzymatic Treatments***					
pectolytic enzymes	hydrolysis of pectins	improving filterability, clarification and stability	reduction of particles size and concentration	yet to be studied	^115^
***IV. Colloidal Stabilization through Additive Use***					
polysaccharides	protein and phenolic colloid coating	preservation of colloidal stability	prevention of further colloidal aggregation; colloidal destabilization and precipitation	color stabilization	^7,121^
synthetic polymers (potassium polyaspartate)	electrostatic interaction and crystal growth inhibition	primarily tartrate stabilization	prevents tartrate precipitation. Can trigger flocculation of unstable coloring matter if not properly prestabilized	potential for increased turbidity if coloring matter is unstable	^131,134,135^

a The techniques are categorized by their primary mechanism of action: (I) physical removal of solids, (II) selective compositional modifications, (III) induced precipitation, and (IV) stabilization through additive use. Each method is assessed based on its winemaking application, effects on colloids, and potential sensory impact.

In practical winemaking, a combination of more than one of these approaches is commonly applied, thus complicating the situation due to the combined effects that they can have on the red wine colloidal state.

Regardless of whether the treatments target actual defects (e.g., haze) or substances potentially causing them over time (e.g., colloids), the common goal remains preserving the properties and quality, including flavor, of red wines during production and from bottling to consumption. Importantly, all interventions share the common necessity of being rapid and minimizing product loss.

### 3.1 Physical Removal of Colloids

The physical removal of colloidal particles and macromolecules represents a fundamental category of red wine stabilization processes. This group includes operations such as racking, filtration, ultrafiltration, and centrifugation, each aimed at enhancing clarity, microbial stability, and overall process efficiency.

#### 3.1.1 Racking and Centrifugation

Among the clarification methods, racking represents the most traditional and simplest approach, relying on the gravitational settling of particles at the tank bottom. According to Stokes’ law, sedimentation rate is strongly correlated with particle diameter, with settling rates dropping by a factor of 10,000 when particle size decreases from 10 μm (e.g., yeast cells) to 0.1 μm (colloidal range).^6,81^ The sedimentation of “true” colloids (defined as particles between 1 and 1000 nm) is typically negligible because the Brownian motion, characteristic of these entities, prevails over the gravitational force.^6,22^ Therefore, spontaneous settling alone is not sufficient to effectively remove colloidal material or prevent long-term instability.^81,82^ To increase the gravitational force, centrifugation can be used. However, although this technique allows for a rapid and efficient removal of large particulate matter (typically >1 μm, such as yeast and cell debris), standard industrial centrifugation is generally insufficient to substantially remove submicron colloidal particles or macromolecules within a time frame compatible with winemaking. Nevertheless, some removal may occur for colloids that are adsorbed onto or entrapped within the larger sedimenting particles.

#### 3.1.2 Filtration and Ultrafiltration

Filtration involves passing the wine through porous media to retain suspended particles and microorganisms, contributing to both visual clarity and microbial control. When using membranes, as in micro- and ultra-filtration, the selectivity of the process largely depends on their porosity, which must be carefully chosen to suit the wine’s composition.

In dead-end filtration, the wine flows perpendicularly through the membrane, allowing particulate matter to accumulate on the surface or within the depth filter. However, particularly in the case of red wines, colloids can rapidly clog membranes, leading to increased pressure, potential wine losses, and operational inefficiencies. The addition of diatomaceous earth could be used to prevent clogging of the support, but significant economic, ecological, and practical limitations exist regarding its handling and disposal.^83^ In general, dead-end filtration of red wines also poses the risk of stripping key components critical for mouthfeel and color stability.^6,81,84^ Notably, the recent literature lacks studies specifically addressing the effect of dead-end filtration on red wine colloids. In contrast, greater attention has been directed toward cross-flow filtration, in which wine flows tangentially along the membrane surface, helping to overcome, at least in part, the problem of membrane clogging. The effectiveness of this process largely depends on membrane material and porosity, which must be chosen to suit the wine’s composition.^85^ Compared to traditional methods using filter aids, cross-flow filtration allows for simultaneous clarification, stabilization, and microbial sterilization under a pressure gradient, significantly reducing winemaking costs and environmental impact by eliminating the need for diatomaceous earth.^84,85^

Membrane filtration processes are primarily distinguished by their pore diameter and separation capability.

Microfiltration (MF) utilizes membranes with pore sizes ranging from 0.1 to 1 μm. In winemaking, a pore size of 0.2 μm is commonly employed for microbial stabilization and final clarification, aiming to achieve turbidity levels generally lower than 1 NTU.^85,86^

Ultrafiltration (UF) represents a distinct category: unlike MF, which primarily targets suspended solids and microorganisms, UF allows for targeted compositional adjustments. Using tighter membranes with pore sizes between 0.01 and 0.1 μm, often characterized by their molecular weight cut-off (MWCO), UF effectively removes macromolecules and colloidal aggregates rather than coarse particulate matter. For specific fractionation strategies in red wines, membranes with MWCOs as low as 10 kDa can be utilized to separate high molecular weight compounds.^85–87^

In both cases, membrane fouling remains a major limitation as it reduces permeation flux, increases operational costs, and may alter the organoleptic properties of wine by retaining sensory-active components.^6,85^ Red wine macromolecules, such as phenolics and polysaccharides, contribute significantly to fouling through their physicochemical interactions with the membrane surface.^88^ These interactions occur in both MF and UF, as they depend on the chemical nature of the membrane material rather than the pore size alone. For instance, membranes containing amide bonds can interact with polar molecules like anthocyanins and tannins through hydrogen bonding,^89^ a phenomenon that can lead to significant color loss even in microfiltration.^85^ On the other hand, polysaccharides can adhere to membranes through both molecular interactions and steric hindrance, particularly when highly branched or characterized by high molecular weight.^86^ Moreover, since these macromolecular colloids influence wine mouthfeel and texture, their removal may affect the sensory profile of the final product.^84–86^ The extent of this impact is dependent on the membrane type, pore size, and filtration conditions. Among polymeric membranes, hydrophilic cellulose acetate has shown superior performance in minimizing the adsorption of both polysaccharides and polyphenolic compounds compared to polyethersulfone) (PES) and polypropylene.^90^

Therefore, membrane selection significantly impacts filtration outcomes and must always be aligned with the specific colloidal composition desired for the wine.^85,91^ To this end, ultrafiltration can be selectively used to remove specific unstable components, such as proteins or complex phenolic aggregates, which could lead to turbidity during shelf life. However, the excessive removal of anthocyanins, polymeric pigments, and tannins remains a concern, as these components are critical for color, structure, and the evolution of mouthfeel during red wine aging.^85–87^ Furthermore, losing “protective colloids” like yeast mannoproteins can be detrimental to long-term colloidal stability.^92^ Finally, the impact on the volatile fraction must be considered, as some aroma compounds are lost when the macromolecular complexes to which they are bound are retained.^93^

For all these reasons, while UF represents an effective tool for protein stabilization in white wines,^94,95^ its application to red wines remains far more challenging. In the latter, the high concentration of colloidal material not only leads to significant operational issues but also poses a high risk of stripping key sensory components, potentially compromising the overall quality and the structural evolution of the wine.^86^

### 3.2 Induced Precipitation

#### 3.2.1 Cold Treatment

Cold stabilization is a widely applied technique in wine production primarily aimed at preventing the formation of tartrate crystals and bottle deposits that can compromise wine clarity and negatively influence consumer perception. Cold stabilization typically involves chilling the wine to near-freezing temperatures to promote the precipitation of potassium bitartrate, which is subsequently removed prior to bottling.^96^ In this situation also, the removal of pigment–tannin complexes that coprecipitate with tartrates has been observed.^8,97^ On the other hand, wine macromolecules are known to prevent the formation of tartrate crystals,^18^ and this is particularly true in red wines, as they are richer in these compounds compared to wines made without skin maceration.^23^

A recent study highlights the role of colloidal and macromolecular components, such as polysaccharides, phenolic compounds, and protein-pigment complexes, in the overall stability of red wines.^5^ Through AF4, coupled with multiple detectors (MALS, dRI, and UV), it was shown that cold stabilization alters the distribution and density of colloidal fractions. The hypothesis is that polysaccharides, identified as high-molecular-weight, low-density colloids, may interact with potassium and tartrate ions, possibly influencing the physicochemical conditions for tartrate precipitation. In parallel, temperature changes during stabilization appeared to affect protein–polyphenol aggregates and pigmented colloids, suggesting their partial removal or structural modification. As most of wine colloidal particles are colored,^2^ their precipitation contributes to the reduction in color intensity, an important practical issue in red winemaking.^97,98^ On the other hand, this phenomenon is also affected by the presence and concentration of polysaccharides that, acting as protective colloids, contribute to the color stability of red wines.^22^

#### 3.2.2 Fining

Due to their very small size and (meta)­stability, colloidal particles often require prior flocculation or aggregation to enable effective sedimentation. To induce this phenomenon, fining agents, also known as clarifiers, are used to promote the adhesion of potentially unstable wine components, such as colloids, onto adsorbing materials. These interactions accelerate the clarification process by promoting the formation of enlarged aggregated particles exceeding colloidal dimensions, which can then be more efficiently removed under gravitational force through settling or centrifugation, or via filtration.^6^ To obtain this effect, adsorption processes occur between the fining agent and target wine components, whereby unstable colloidal particles adhere to the surface of the fining material. This adsorption is driven by multiple physicochemical interactions, including van der Waals forces, hydrogen bonding, and electrostatic and hydrophobic interactions.^6,81^

Proteins of various origins have long been used as clarifying agents in red winemaking due to their hydrophobic character, which enables them to interact with and bind phenolic compounds and polymeric pigments involved in colloidal instability.^6,81,99–101^ Indeed, the interaction of tannins with these proteins results in the formation of insoluble particles that can be physically removed, thereby improving colloidal stability while also modifying wine astringency and/or bitterness. Protein fining agents could also affect more complex colloidal structures containing tannins, although this aspect has yet to be investigated.

Traditionally used protein-based fining agents include gelatin, casein/K-caseinate, egg albumin, and isinglass, even if, in recent years, plant-derived proteins have also been proposed and many, such as potatoes and pea proteins, have also become commercially available because they avoid the allergen-related health concerns of animal proteins address consumer preferences.^102–104^ Among protein fining agents, gelatin is the richest in proline, which is the amino acid most involved in reacting with wine tannins, leading to the effective flocculation of the formed complexes.^81,105^ Although monomeric anthocyanins exhibit lower direct affinity for proteins compared to tannins, several studies have reported a negative impact on wine color following protein fining.^99,105,106^ This reduction in color intensity is generally attributed to the removal of proanthocyanidins that stabilize anthocyanins via copigmentation,^107^ or the interaction between protein finings and colored polymeric pigments leading to their removal,^108^ with consequences on colloidal particles in which polymeric pigments have been shown to be a constituent.^1,2^

Another common fining agent is bentonite, a clay classically used for white wines to prevent protein haze formation,^109^ but increasingly applied also in red winemaking for clarification and colloidal stabilization purposes.^110^ Bentonite carries a negative charge at wine pH as a result of its layered structure and exchangeable sodium or calcium cations. This allows bentonite to bind and remove positively charged wine proteins through cation exchange,^111^ while some phenolic compounds can be removed indirectly when associated with these proteins.^3^ However, compared to protein-based fining agents, bentonite tends to have a stronger impact on monomeric anthocyanins, specifically those present in the positively charged flavylium form. In addition, small and large polymeric pigments can also be removed by bentonite, as recently demonstrated for a red wine characterized by a medium-low degree of colloidal instability.^32^ However, the direct adsorption often results in a more significant negative effect on the wine’s color intensity than protein finings.^106,112^ Therefore, in addition to its clarification effect, its use is advised to remove unstable colloids such as proteins and polymeric pigments.^6^

Other fining agents used in red winemaking include polyvinylpolypyrrolidone (PVPP) and polyvinylpyrrolidone (PVP), both derived from vinylpyrrolidone. Given their polyamide nature, these agents exhibit selective adsorption of phenolic compounds.^81,105^ However, in red wine production, they are more commonly employed to mitigate phenolic off-odors, particularly those caused by ethylphenols, rather than to remove unstable polyphenols responsible for turbidity.^103,113^ Indeed, this was experimentally confirmed in a recent study showing that fining with PVP did not result in colloidal stabilization of two red wines.^32^

Taken together, available data indicate that the removal of the colloidal matter from the red wines to obtain their stabilization is a complex issue. This process strongly depends on the physicochemical properties of the fining agents, their dosage, and the treatment conditions, which mainly affect phenolics crucial for wine color, mouthfeel, and aroma. In addition, the composition of the red wine itself significantly affects the effectiveness and outcomes of the process. This effectiveness is strongly influenced not only by the concentration and interactions of wine components but also by the wine’s pH, ionic strength, ethanol content, the presence of protective colloids, and the temperature during treatment,^6,81,82,110^ confirming the assumption that each wine needs a tailored fining protocol.

### 3.3 Enzymatic Treatments

An interesting approach for the selective modification of wine colloids is enzymatic treatment, which is commonly used in red wine production to assist the maceration process, improve filterability and clarification, and positively influence color and aroma.^114^ Despite the potential of enzymatic treatments, typically due to their specificity of action, their capacity to modify the colloidal status of red wines has not been fully explored. In this context, only the impact of pectolytic enzymes on the concentration of colloidal particles has been investigated.^115^ The results confirmed that pectinase treatments can reduce the concentration of colloidal particles in red wines by hydrolyzing high-molecular-weight pectins. These polysaccharides often act as protective colloids; their degradation removes the steric stabilization that keeps particles in suspension, thereby facilitating their aggregation and subsequent removal during clarification.^115^

In contrast, β-glucanases may influence wine colloids by promoting the hydrolysis of yeast cell wall β-glucans,^116^ thereby enhancing the release of macromolecules, especially mannoproteins that are known to interact with tannins and other macromolecules, contributing to steric stabilization and modulating aggregation phenomena.^117^ Consequently, β-glucanase treatments may shift the colloidal equilibrium by increasing the concentration of yeast-derived polysaccharides involved in colloidal structures.

In addition to polysaccharides, proteins play a key role in colloid formation^1^ and, possibly, stability. Therefore, the enzymatic degradation of grape proteins could probably influence the colloidal status of wine. However, scientific studies specifically examining the impact of proteases on the colloidal stability of red wines are currently lacking. Proteolytic enzymes, such as aspergillopepsins, have proven effective in preventing protein haze in white wines,^118^ and are now approved for enological use.^119^ While commercial preparations are already marketed to manage red wine stability, further research is certainly needed to scientifically validate their effect on red wine colloid composition and behavior.

### 3.4 Colloidal Stabilization through Additive Use

This category includes strategies aimed at preventing the growth and aggregation of colloidal particles through the use of exogenous additives, primarily polysaccharides, which function as “protective colloids.” These additives are used to mimic or enhance the stabilizing effect of endogenous polysaccharides (such as those released during maceration or yeast autolysis) by inhibiting the flocculation of unstable particles without requiring their physical removal.

The most established additives in this context are protective polysaccharides or gums, as steric stabilizers. Several studies have explored their addition to manage red wine stability.^6,21,120^ However, the effectiveness of these treatments is complex and highly dependent on both the structural characteristics of the additive and the specific wine matrix.^7^ For instance, Bosso et al.^32^ demonstrated that the stabilization achieved by adding exogenous mannoproteins and gums varies significantly across different red wine varieties, suggesting that the initial phenolic content dictates how well those additives can prevent aggregation. Similarly, Zhai et al.^21^ confirmed that the long-term stability of three red wines was modulated by the interaction between the structures of the added polysaccharides (mannoproteins and arabinogalactans) and the native phenolics and polysaccharides present in different wines. The importance of molecular structure is further supported by recent research showing that the molecular weight and degree of esterification of pectic fragments are decisive factors explaining why specific additives, such as*Acacia Senegal*gum, are particularly effective as stabilizers.^121^ This confirmed that*Acacia senegal*gum acts as a protective colloid against the precipitation of coloring matter, its effectiveness being due to its specific glycosylation rate and protein content, which modulate its hydrophobicity and its ability to shield unstable pigments.^31^

In addition to these polysaccharides, the possible re-evaluation of carboxymethylcellulose (CMC) in red wine systems, particularly in combination with other stabilization strategies,^122^ may be of interest. Although CMC is widely used for tartaric stabilization,^123^ its interactions with phenolic compounds are well-known to negatively affect color and colloidal equilibrium,^124,125^ making this additive currently not allowed for use in red wine.

Among other polysaccharide-based additives, chitosan has been extensively investigated in winemaking, primarily for microbial control and metal removal.^126,127^ Due to its cationic nature, chitosan can interact with negatively charged wine components, including proteins, colloidal particles, and, to some extent, phenolic compounds, promoting flocculation and clarification phenomena. These effects are mainly attributed to electrostatic interactions that destabilize colloidal systems and favor aggregation processes.^128^ However, its role in modulating the colloidal stability of red wines remains only partially understood.

In contrast, polysaccharides derived from seaweeds, such as carrageenan,^129^ have been explored mainly as alternative fining agents. These compounds have demonstrated the ability to bind proteins in white wine systems,^130^ a property that suggests a potential influence on the colloidal behavior of red wines. However, their specific role in the stabilization of red wine colloids has yet to be investigated.

Beyond polysaccharides, synthetic polymers such as metatartaric acid^131^ and, more recently, potassium polyaspartate (KPA) have emerged as tools for tartrate stabilization.^132^ Metatartaric acid acts primarily as a crystal growth inhibitor and, due to its limited and non-specific interactions with wine macromolecules, should have negligible effects on the colloidal stability of red wines. Similarly, KPA primarily acts as a crystal growth inhibitor through electrostatic interactions, but it also interacts with the wine macromolecules and unstable coloring matter, potentially triggering the flocculation of pigment–tannin complexes.^133^ However, if the wine is previously stabilized, these effects can be avoided.^134,135^ Therefore, in red wines, the use of KPA requires careful evaluation, as it may modulate the colloidal equilibrium of pigments.

To consolidate the information presented above, Table 1 summarizes the main winemaking techniques used for red wine colloid management, categorized by their mechanism of action and evaluated in terms of winemaking application, effects on colloidal stability, and potential sensory impacts.

## 4 Conclusions and Future Directions

Achieving colloidal stability in red wine is critical for ensuring quality, shelf life, and consumer acceptance, as it preserves both visual clarity over time and the wine’s sensory profile. However, achieving this stability without compromising the quality of the wine, especially for red wines destined for aging, remains a key challenge for winemakers, also because a deep knowledge of red wine colloid structure and behavior is lacking. This review has explored recent advances in understanding red wine colloidal nature by summarizing the current knowledge on the constituents, the structures, and functionality of colloidal particles in red wines. In recent years, significant progress in this field has been driven by the application of advanced analytical and fractionation techniques, including methods aimed at the characterization of colloidal size distribution, composition, and interactions, such as DLS and spectroscopic approaches. Actually, among these advanced techniques, AF4 has emerged in recent years as allowing more relevant advancements. However, despite this progress, the nature of red wine colloids, their behavior, and the factors governing it still need further investigation, also for practical reasons. Indeed, the management of the colloidal status of red wine remains critical for winemaking, and it appears that no single technique can fully stabilize wine colloids as each strategy presents both advantages and drawbacks. The inherent complexity of wine and the dynamic nature of colloidal aggregation further complicate the identification of a universally optimal approach. In this context, future research should prioritize the development of more selective and rational stabilization strategies. For example, specific enzymatic approaches may be adopted in order to modulate the level of colloid-forming molecules in a more targeted way compared with traditional fining or stabilization treatments. For example, the use of enzymes to facilitate the extraction of polysaccharides from both yeast and grapes, or to modify the protein profile of wines, may offer new tools to manage structures, and consequently behavior, of colloids in red wines. However, their role in red wine systems remains poorly understood and requires further investigation to assess their effectiveness and potential unintended impacts on wine composition and sensory properties. More broadly, future research should explore these aspects, exploiting the recent advancements in both colloidal and wine science, improving the understanding of the mechanisms and timing of colloidal particle formation, their evolution during aging, and their response to key variables in terms of interactions and stability.

## Data Availability

The data presented in this study are available upon request from the corresponding author.
